# Experimental antibiotic treatment identifies potential pathogens of white band disease in the endangered Caribbean coral *Acropora cervicornis*

**DOI:** 10.1098/rspb.2014.0094

**Published:** 2014-08-07

**Authors:** M. J. Sweet, A. Croquer, J. C. Bythell

**Affiliations:** 1 Biological Sciences Research Group, University of Derby, Kedleston Road, Derby DE22 1GB, UK; 2 School of Biology, Molecular Health and Disease Laboratory, Newcastle University, Devonshire Building, Newcastle upon Tyne NE1 7RU, UK; 3 Departamento de Estudios Ambientales, Universidad Simón Bolívar, Ap. 89000 Caracas, Venezuela; 4 Research Office, University of the South Pacific, Suva, Fiji

**Keywords:** coral, disease, treatment, antibiotics, *Philaster*, *Vibrio*

## Abstract

Coral diseases have been increasingly reported over the past few decades and are a major contributor to coral decline worldwide. The Caribbean, in particular, has been noted as a hotspot for coral disease, and the aptly named white syndromes have caused the decline of the dominant reef building corals throughout their range. White band disease (WBD) has been implicated in the dramatic loss of *Acropora cervicornis* and *Acropora palmata* since the 1970s, resulting in both species being listed as critically endangered on the International Union for Conservation of Nature Red list. The causal agent of WBD remains unknown, although recent studies based on challenge experiments with filtrate from infected hosts concluded that the disease is probably caused by bacteria. Here, we report an experiment using four different antibiotic treatments, targeting different members of the disease-associated microbial community. Two antibiotics, ampicillin and paromomycin, arrested the disease completely, and by comparing with community shifts brought about by treatments that did not arrest the disease, we have identified the likely candidate causal agent or agents of WBD. Our interpretation of the experimental treatments is that one or a combination of up to three specific bacterial types, detected consistently in diseased corals but not detectable in healthy corals, are likely causal agents of WBD. In addition, a histophagous ciliate (*Philaster lucinda*) identical to that found consistently in association with white syndrome in Indo-Pacific acroporas was also consistently detected in all WBD samples and absent in healthy coral. Treatment with metronidazole reduced it to below detection limits, but did not arrest the disease. However, the microscopic disease signs changed, suggesting a secondary role in disease causation for this ciliate. In future studies to identify a causal agent of WBD via tests of Henle–Koch's postulates, it will be vital to experimentally control for populations of the other potential pathogens identified in this study.

## 1. Introduction

Coral reefs and other tropical marine systems have declined in health in recent decades, owing to a variety of local and regional environmental impacts in addition to the effects of climate change. These impacts threaten the fundamental ecological functions of coral reefs [1] as well as the coastal protection, tourism, biodiversity, fisheries production and other ecosystem services that they provide [2].

Many reef coral diseases have emerged in the past 30–40 years, several of which have caused significant regional-scale ecological impacts [3–5]. For example, *Acropora* species were formerly the dominant ‘bioengineering’ species on shallow and mid-depth zones over most of the Caribbean. Shallow (0–6 m depth) reefs were typically dominated by *Acropora palmata*, whereas *Acropora cervicornis* was commonly a dominant species at 6–9 m depth [6]. These species are now relatively rare and recently both species have been listed as critically endangered on the International Union for the Conservation of Nature Red list. In consequence to this loss, the physiographic reef zones they had previously constructed have been eliminated from all but a handful of reefs in the region [7]. It is widely regarded that this decline can be attributed to the emergence of a particularly aggressive coral disease, white band disease (WBD), first observed in the early 1970s [8–10].

As with many coral diseases, the causal agent of WBD has not been definitively identified, and there is some confusion over the two types of WBD that have been described (type I and II). WBD type II is differentiated from WBD type I by a variable band of bleached tissue that precedes the tissue lesion [11]. A causal agent for WBD type II has previously been proposed as *Vibrio charchariae*, which is a synonym of *Vibrio harveyi* [11]; however, the reisolation and definitive identification of the proposed agent from experimental inoculations has never been documented. With regard to WBD type I, Pantos & Bythell [12] failed to detect any *Vibrios* in diseased tissues using culture-independent techniques [12], however, they did show a suite of α-proteobacteria related to *Roseobacter* to be present in WBD type I, but absent in healthy corals. These were similar to a group of bacteria previously detected exclusively in diseased tissues of other coral diseases (white plague and black band disease). By contrast, Casas *et al.* [13] failed to detect any specific pathogens in WBD using culture-independent techniques and suggested that there might be a non-pathogenic cause of this disease. However, recently, a study by Kline & Vollmer [14] used homogenates of diseased tissue to demonstrate that WBD is, in fact, a transmissible disease attributable to a 0.22–0.45 µm filterable fraction that was susceptible to antibiotic treatment. They concluded that the causal agent is therefore likely to be a Gram-positive bacterium. However, their study did not involve a comprehensive analysis of the microorganisms involved and therefore the pathogen remains unknown. In this study, a broader set of antibiotics were used to treat WBD type I diseased corals (*A. cervicornis*) directly, together with a comprehensive screening of the microbial community (bacteria, archaea and ciliates) associated with the coral using culture-independent molecular techniques to detect potential pathogens and narrow the range of potential agents that could be further tested for causality using challenge experiments.

## 2. Results

**Figure 1.**
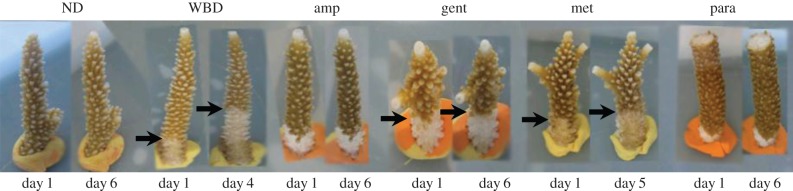
Photographs of healthy, non-diseased (ND) corals and those with signs of white band disease (WBD) both controls and treated corals. The experiment was run for 6 days, during which the healthy corals showed no signs of lesion development or other visible signs of stress. The lesion of untreated corals showing signs of white band disease Type 1 (WBD) continued to progress, while those treated with ampicillin (amp) and paromomycin sulfate (para) arrested lesion progression. (Online version in colour.)

A range of antibiotics were used to treat corals showing signs of WBD type I with the aim to detect potential pathogens and reduce the overall range of potential causal agents highlighted in previous studies. We found a significant interaction between time and treatment (repeated-measures ANOVA, *F*_120_ = 8.03, *p* = 0.0001), on lesion progression rate (table 1), indicating that the effect of each antibiotic on WBD progression was different for the period of observation. All apparently healthy, non-diseased (ND) corals survived the 6 days of the experiment and appeared normal at the end of the experiment (figure 1). Disease progression continued in all the diseased corals not treated with antibiotics throughout the duration of the experiment (figure 1 and table 1), and the advance rates of the disease lesion of these untreated corals (from 0.9 ± 0.10 to 3.4 ± 0.40 cm d^−1^) were within the range reported for WBD in the field (0.2–4 cm d^−1^) [15]. Initial experiments on healthy corals treated with the four types of antibiotic used in this study showed no visible adverse effects of the treatments.

**Table 1.** Mean lesion progression rate (cm d^−1^) of white band diseased coral nubbins during the antibiotic experiment. (Time 0 represents lesion progression rate in the 12 h prior to the start of the experiment confirming that all lesions were advancing prior to treatment. ND, non-diseased corals kept under experimental conditions as controls. No disease lesions developed in these controls. WBD, white band diseased corals untreated with antibiotic. These lesions continued to progress throughout the experiment and samples were collected before the whole coral nubbin had died (see + for collection points). Amp, ampicillin-treated white band diseased corals. Lesion progression was immediately halted under this treatment. Gent, gentamicin-treated white band diseased corals. Lesions in this treatment continued to progress throughout the experiment, but all nubbins survived to the end of the experiment and were sampled at 144 h. Met, metronidazole-treated white band diseased corals. Lesions in this treatment continued to progress throughout the experiment, and samples were collected before the nubbins were completely killed (see + for collection points). Para, paromomycin-treated white band diseased corals. Lesion progression halted after 24 h in this treatment. Plus symbols show time points when an individual nubbin was sampled, either when <1 cm of tissue was left on the coral nubbin or at the end of the experiment. Data are means ± s.e. (*n* = 6 initially).)

time (h)	ND	WBD	amp	gent	met	para
0	0	0.9 ± 0.10	0.9 ± 0.10	0.8 ± 0.23	0.9 ± 0.20	0.9 ± 0.2
24	0	0.9 ± 0.07	0	0.6 ± 0.19	0.6 ± 0.19	0.7 ± 0.45
48	0	1.2 ± 0.08	0	0.8 ± 0.28	1.4 ± 0.3	0
72	0	1.5 ± 0.20	0	1.4 ± 0.53	1.8 ± 0.55 ++	0
96	0	3.4 ± 0.40 ++++	0	1.7 ± 0.7	2.3 ± 0.7	0
120	0	++	0	1.9 ± 0.8	1.9 ± 0.1 +++	0
144	0		0	1 ± 0.4	++	0
	++++++		++++++	++++++		++++++

### (a) Identification of potential pathogens

There was little variation apparent in the bacterial diversity of replicate samples of the same treatment, as determined by denaturing gradient gel electrophoresis (DGGE) analysis (electronic supplementary material, figure S1); therefore, only a subset (*n* = 3) of randomly selected samples were used for detailed clone library analysis. There were significant differences in 16S rRNA gene bacterial diversity in both the clone libraries (*n* = 3 per treatment) and the DGGE (*n* = 6 per treatment; analysis of similarity (ANOSIM *R* = 1, *p* = 0.029 and *R* = 1, *p* = 0.001, respectively) between all treatments (electronic supplementary material, table S1). Despite metronidazole-targeting protozoans, this treatment also had a significant effect on the bacterial communities (electronic supplementary material, table S1).

Fifteen prokaryote (bacteria and archaea) ribotypes were identified in this study as potential pathogens of WBD, being consistently present in all samples of corals displaying WBD, but absent in ND samples. These were ribotypes related to: *Oceanicola* (KC736995), *Sandarakinotalea* (KC736996), *Anaeroplasma bactoclasticum* (KC736998), *Parachlamydia acanthamoebae* (KC737010), *Photobacterium aplysia* (KC737015), *Comamonas* (KC737017), *Halobacteria* (KC737020), *Asteroleplasma* (KC737022), *Vi. charchariae* (KC737024), *Lactobacillus suebicus* (KC737026), *Roseovarius crassostreae* (KC737031), *Bacillus* (KC737032), *Cyclobacterium* (KC737035) and an unidentified β-proteobacteria (KC737036; electronic supplementary material, table S1). An additional ribotype, an archaea, *Pyrobaculum* (KC737045) was present only in low frequency in one of the three samples of ND corals and frequent in all three diseased samples and was therefore also considered a potential pathogen. The *Vi. charchariae* ribotype has previously been identified (100% sequence similarity) as a pathogen in WBD type II [11], the *Ri. crassostreae* ribotype (100% similarity) was highlighted as a potential pathogen of WBD type I [12] and the *Bacillus* species (100% similarity) was identified as a possible pathogen of WBD in acroporas from Indonesia [16].

**Figure 2.**
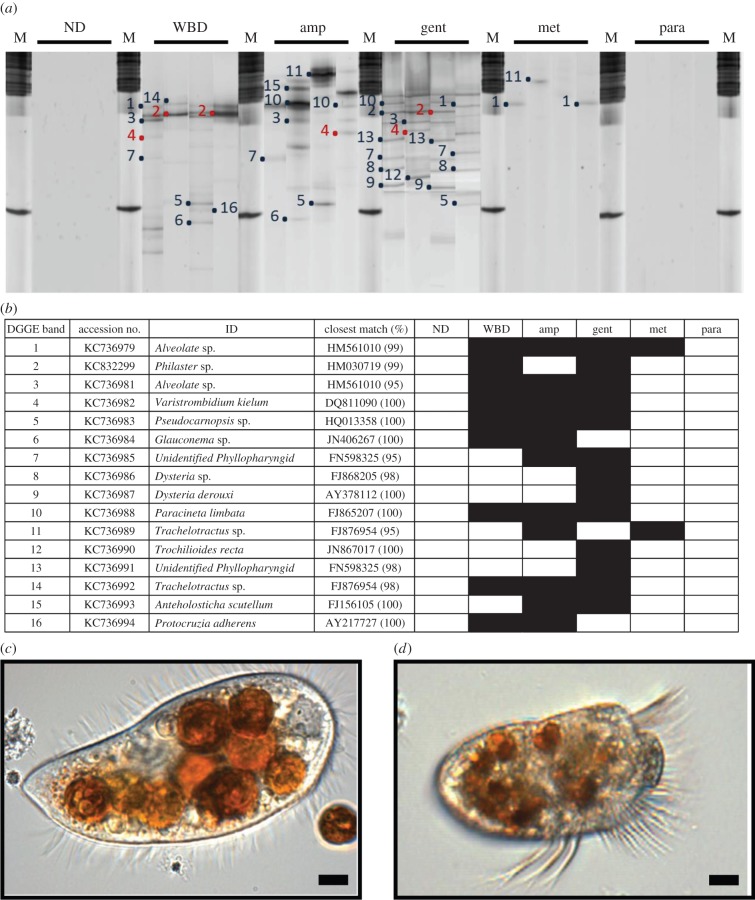
(*a*) Representative denaturing gradient gel electrophoresis (DGGE) profiles obtained using ciliate-specific primers of: non-diseased (ND); white band diseased (WBD); ampicillin treated (amp); gentamicin-treated (gent); metronidazole-treated (met) and paromomycin-treated (para) corals; (*b*) summary table showing presence/absence of specific ciliates in the different samples, (*c*) light micrograph of the histophagous ciliate *Phialster lucinda* (KC832299); and (*d*) light micrograph of the other histophagous ciliate *Varistrombidium kielum* (KC736982). Ingested coral symbiotic algae clearly visible. Scale bars represent 10 µm. (Online version in colour.)

There was a diverse community of ciliates associated with WBD samples, but ciliates were not detected in any of the ND samples (figure 2). Ciliate ribotypes present in samples with WBD were related to: two *Alveolate* sp. (KC736981 and KC736979), a *Pseudocarnopsis* sp. (KC736983), a *Glauconema* sp. (KC736984), *Paracineta limbata* (KC736988), a *Trachelotractus* sp. (KC736992), *Protocruzia adherens* (AY217727), *Philaster lucinda* (KC832299) and *Varistrombidium kielum* (KC736982). The latter two histophagous ciliates have previously been found associated with diseased corals (white syndrome; WS) in the Pacific [17]. *Philaster lucinda* (99% sequence similarity) was consistently present in all samples of WBD and has similarly been observed consistently in WS in the Pacific [17]. As previously observed in WS, this highly active ciliate was present in dense, mobile population masses and observed to burrow under the lesion boundary and consume intact coral tissues in all WBD samples analysed in this study (electronic supplementary material, video S1). The *Varistrombidium* species (100% sequence similarity to that present in WS in the Pacific) is also histophagous (observed ingesting the coral tissues and engulfing the symbiotic algae), but was not consistently observed in all diseased samples. No ciliate other than *Phi. lucinda* was consistently observed in all samples of the disease, and it is therefore likely that the other species observed are secondary invaders.

### (b) Identification of candidate primary pathogens by experimental antibiotic treatment

In specimens initially displaying an actively progressing WBD lesion, two antibiotic treatments, ampicillin and paromomycin sulfate, arrested the advance of the lesion in all samples (figure 1 and table 1; *n* = 6 coral fragments per treatment). The disease lesion continued to progress in diseased corals treated with gentamicin and metronidazole (figure 1 and table 1). Of the 15 prokaryote candidate pathogens identified above, seven were reduced to undetectable levels by the gentamicin treatment that failed to arrest the progression of the WBD lesion; (*Oceanicola* (KC736995), *Sandarakinotalea* (KC736996), *Pa. acanthamoebae* (KC737010), *Pho. aplysia* (KC737015), *Comamonas* (KC737017), *Cyclobacterium* (KC737035) and the β-proteobacteria (KC737036)). Similarly, the ciliate *Philaster lucinda* was reduced to undetectable levels by the metronidazole treatment that failed to arrest the lesion progression. It was conversely eliminated in both the ampicillin and paromomycin treatments. These eight candidate pathogens are therefore unlikely to be primary pathogens. Of the eight remaining candidate pathogens, five were not eliminated either by the ampicillin treatment, the paromomycin sulfate treatment, or both, which arrested the WBD lesion progression (*Anaeroplasma bactoclasticum* (KC736998), *Halobacteria* (KC737020), *Asteroleplasma* (KC737022), *R. crassostreae* (KC737031) and *Pyrobaculum* (KC737045)). Thus, three of the potential pathogens identified in this study: *V. charchariae* (KC737024), *L. suebicus* (KC737026) and the *Bacillus* sp. (KC737032) remain as potential primary pathogens of WBD.

### (c) Association of potential pathogens with tissue pathogenesis

**Figure 3.**
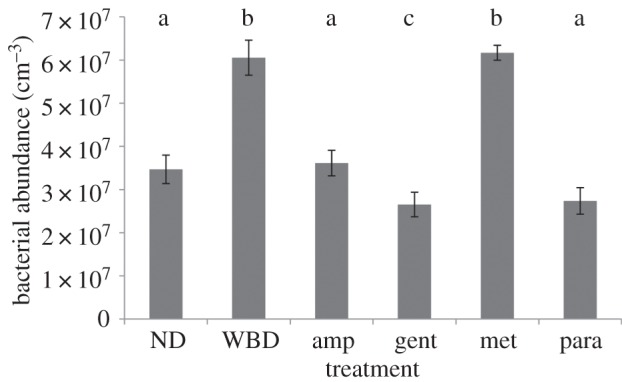
Total bacterial abundance of non-diseased tissues (ND), corals with white band disease (WBD) and diseased corals treated with paromomycin sulfate (para), ampicilin (amp), gentamicin (gent) and metronidazole (met). Letters above the bars (a, b and c) show which treatments showed significant differences (Tukey's HSD *post hoc* tests).

**Figure 4.**
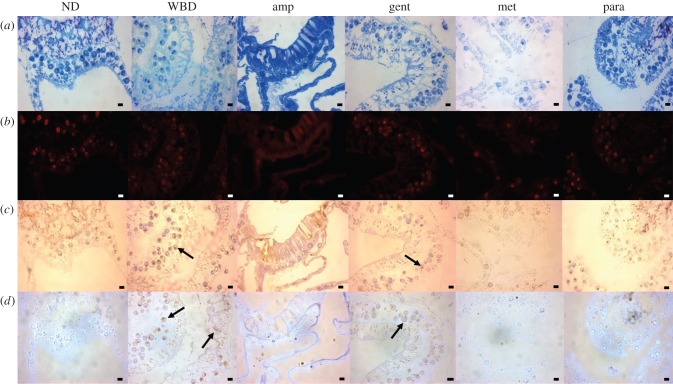
Representative histological sections from each treatment at day 6 of the experiment for non-diseased tissues (ND) and those treated with paromomycin sulfate (para), ampicilin (amp) and gentamicin (gent). The white band diseased (WBD) tissues and those treated with metronidazole (met) were sampled before complete tissue loss occurred, on days 2, 3, 4 and 5 (table 1). In all cases (except that of ND tissues), the samples were taken at the disease lesion interface, within 1 mm of the lesion boundary. (*a*) Sections stained with Toluidine blue. Tissues appear indistinguishable from healthy samples in the diseased (WBD) samples and in all treatments except for the Met treatment, in which extensive tissue fragmentation can be seen. (*b*) Fluorescence *in situ* hybridization (FISH) probed with EUBMIX, giving a comprehensive ‘eubacterial’ detection as visualized by red (CY3) fluorescence. In this case, few if any bacterial-sized fluorescent particles can be identified in the sections, with red fluorescence attributed to autofluorescence of symbiotic algae and nematocysts. (*c*) *In situ* end labelling (ISEL), programmed cell death assay. Little or no probe binding (indicated by red-brown staining) could be detected in ND tissues and those treated with amp and para. Positive ISEL staining was observed in diseased tissues (WBD) and gent-treated samples (arrows). (*d*) Nigrosin (Nig) staining, which targets necrotic tissues. Brown-stained (necrotic) host tissues and symbiotic algae were observed in diseased (WBD) and gent treatments (arrows), but not in any other samples. Para- and amp-treated tissues appeared indistinguishable from healthy tissue sections in all cases. Scale bars represent 10 µm. (Online version in colour.)

There was a significant difference in bacterial abundance between tissues (ANOVA *R* = 0.87 *p* = 0.001; figure 3). Specifically, there was a significant increase in total bacterial abundance between ND and WBD diseased tissues (*p* < 0.0001; figure 3). However, there was no large bacterial mass or evidence of widespread tissue necrosis and/or apoptosis in histological sections (figure 4), indicating that the bacterial population build-up probably occurred in the surface mucus layer, external to the tissues, which would be lost during routine histological processing. However, sections stained with nigrosin indicated some cellular necrosis within both the host tissues and symbiotic algae of WBD samples (figure 4). Necrosis was present at low levels in the peripheral surface of the epidermis and in localized deeper pockets (figure 4). Furthermore, a similar positive staining was associated with tissues stained with *in situ* end labelling (ISEL) indicating simultaneous apoptosis occurring within the necrotic tissues (figure 4). In corals treated with ampicillin, gentamicin and paromomycin sulfate, the tissues appeared normal under the general stain toluidine blue (figure 4), whereas corals treated with metronidazole showed extensive tissue fragmentation, but without any evidence for cellular necrosis or apoptosis (figure 4). Cellular necrosis, as detected by nigrosin staining, and apoptosis, as detected by ISEL, were observed in the gentamicin treatments, following a similar pattern to that of the untreated WBD tissues with low levels of staining in the peripheral epidermis.

Bacterial abundance in corals treated with ampicillin and paromomycin sulfate (3.61 × 10^7^ cm^−3^ and 2.73 × 10^7^ cm^−3^, respectively) returned to similar levels recorded in non-diseased tissues (3.46 × 10^7^ cm^−3^; figure 3). Furthermore, there was no significant difference in bacterial abundance between WBD tissues and those treated with metronidazole (*p* = 0.12; figure 3), although bacterial species composition was affected (electronic supplementary material, table S1).

## 3. Discussion

We show here that WBD is amenable to treatment with either ampicillin or paromomycin sulfate and therefore support previous studies [14] that have used the application of filtrate to conclude that WBD is caused by microorganisms rather than by physiological stress. This study also shows that WBD, as previously shown in this disease [12], and other diseases such as WS [17–19], white plague [20–25] and black band disease [26,27] is a polymicrobial disease associated with a number of specific microorganisms that are consistently associated with diseased samples but absent, or undetectable, in healthy ones. We identify a suite of 16 such specific associates (14 bacteria, one archaea and one ciliate) as potential pathogens of WBD. One of these, the ciliate *Phi. lucinda* (KC832299), has recently been shown to be consistently associated with the coral disease WS in the Pacific [17] and within aquaria [28], which all have identical visible and histopathological disease signs, namely the advancing band of cleared skeleton immediately adjacent to visibly normal tissues (table 2). In all cases, the ciliate has been observed to ingest visibly intact coral tissues and to engulf and digest the coral endosymbionts. Furthermore, what was probably the same ciliate species, has also been observed to digest entire coral spats [29], although at the time it was misidentified as *Helicostoma nonatum*. This histophagous activity and the consistent, specific association of *Phi. lucinda* with the two diseases indicates that: (i) the ciliate is an active pathogen, and (ii) that the two diseases are probably synonymous. However, selective elimination of this pathogen using the antibiotic metronidazole failed to arrest disease lesion progression in controlled experiments, indicating that the *Philaster* (KC832299) ciliate is unlikely to be a primary pathogen of WBD. However, histological analysis showed that the histopathology of the disease changed in the metronidazole treatment. This result is consistent with this ciliate being a secondary pathogen which nonetheless contributes to the typical pathogenesis of WBD and WS [17]. We also show that another histophagous ciliate, *V. kielum* (KC736982), also associated with WS in Pacific corals, is present in WBD, but is not consistently associated with all cases of the disease. This and a diverse community of other ciliates are non-specific associates, likely to be secondary invaders of WBD. Observations from these studies have led us to the conclusion that the visible disease signs of WBD and WS [17], can largely be attributed to this ciliate assemblage and other protozoans and micro-metazoans feeding on both the intact tissues (*Philaster lucinda* and *Va. kielum*) and tissue detritus at the edge of the lesion (many species; figure 2).

**Table 2.** Summary showing the main effects of treatments and associated potentially pathogenic bacteria (*Vibrio carchariae, Lactobacillus* sp., *Roseovarius crassostreae* and a *Bacillus* sp.) and histophagous ciliates (*Philaster lucinda* and *Varistrombidium* sp.) identified within the study.

	ND	WBD	amp	gent	met	para
progressive lesion	−	+	−	+	+	−
tissue necrosis	−	+	−	+	−	−
apoptosis	−	+	−	+	−	−
tissue fragmentation	−	−	−	−	+	−
*Vibrio carchariae*	−	+	−	+	+	−
*Lactobacillus* sp.	−	+	−	+	+	−
*Roseovarius crassostreae*	−	+	+	+	+	−
*Bacillus* sp.	−	+	−	+	+	−
*Philaster lucinda*	−	+	−	+	−	−
*Varistrombidium* sp.	−	+	+	+	−	−
*in vitro* effect on ciliates	NA	NA	−	−	+	+

Fifteen potential prokaryote (bacterial and archaeal) pathogens were also identified in this study that were consistently associated with all samples of disease and absent from ND samples. The selective elimination of different groups of these prokaryotes by antibiotic treatment indicated that most of these are likely to be secondary pathogens or secondary invaders, despite their specific and consistent association with the disease. Three of the identified candidate bacterial pathogens, *Vi. charchariae* (KC737024), *L. suebicus* (KC737026) and the *Bacillus* sp. (KC737032) could not be eliminated by selective antibiotic treatment and remain as potential primary pathogens of WBD. Two of these have previously been implicated as causal agents in this and similar ‘white diseases’ in other locations and host species, including *Vi. charchariae* in WBD type II [11], and the *Bacillus* sp. in WBD in Indonesian acroporas [16].

By combining a culture-independent analysis with selective antibiotic treatment, we were able to identify a diverse pool of candidate pathogens involved in WBD. While several authors [30,31] have suggested that a wide range of normally commensal, non-specific microorganisms may become pathogenic under the right conditions, such as environmental stress-related reductions in immunity [32], these candidate pathogens were not detected in ND tissues (using the specific techniques in this study), suggesting that these potential pathogens are not a normal part of the commensal community. Most of these candidate pathogens appear to have no significant role in pathogenesis, whereas three potential bacterial primary pathogens and one specific ciliate secondary pathogen were identified. A key question is whether a combination of more than one of these three potential primary bacterial pathogens is required to maintain the disease state, although all three plus the ciliate consistently co-occurred in the disease in nature. Regardless, results from this study strongly suggest that at least one from this candidate group cause a systemic infection resulting in compromised health indicated by limited cell necrosis and increased apoptosis (both in the host tissues and the symbiotic algae) in advance of the disease lesion (figure 3). Interestingly, an increase in apoptosis has been identified previously in corals showing signs of WS [33], again highlighting similarities between these two diseases. The ciliate *Phi. lucinda* contributes to the pathology of the disease yet appears not to be a primary causal agent. Although these results highlight potential pathogens of WBD, challenge experiments such as Henle–Koch's postulates would be required to definitively prove causation. However, such challenge experiments must also control for the effects of the treatments on naturally occurring populations of these and other potential pathogens of WBD.

Although antibiotic treatments could be used as a potential cure for WBD in the field, extreme care would need to be taken as many microbes are known to develop resistance to antibiotics [34,35]. Furthermore, such treatments might have unwarranted effects on other host–microbe interactions in the natural environment. It would be unfeasible and unethical to apply antibiotic treatment at the regional and global scale of coral disease zoonoses, but it would probably be effective to use ampicillin or paromomycin sulfate treatment in specific circumstances where collateral effects could be minimized, for example in aquarium treatments.

## 4. Methods

*Acropora cervicornis* showing signs of WBD (type I) were monitored to show signs of progression. Only those with advancing lesions were used in the experiment. *N* = 2, 5 cm^2^ coral nubbins showing signs of WBD (type I) were placed in three individual aquaria per treatment. Therefore, *n* = 6 replicate coral nubbins were used for each treatment. All corals were maintained in the aquarium for 24 h prior to the start of the experiment to allow for acclimatization and to confirm lesion progression in diseased nubbins. Four types of antibiotics were used in treatments to determine their effects on the diseased corals; ampicillin, gentamicin, metronidazole and paromomycin sulfate. 100 µg ml^−1^ was used for all four antibiotics after preliminary laboratory trials on both bacteria and healthy corals. The antibiotics were added directly into tanks filled with 3 l of seawater collected from the original location of the corals. Repeat dosage was dissolved in 1.5 l of seawater every 12 h, and half the water in the experimental tanks was replaced with the new water. *n* = 6 corals were used per treatment (*n* = 2 per tank, three tanks). One set with WBD were left untreated in the tanks and sampled before all the tissue had been lost. Healthy corals were also collected and held in the aquaria for the duration of the experiment to address any tank effects on the health of the corals. Prior to the onset of the main experiment, healthy corals were also treated with the antibiotics at the same dose rates used within the experiment to ensure that the antibiotics were having no adverse effect on the corals. All these treatments survived to the end of the experiment with no visual appearance of tissue deterioration or discoloration.

Ampicillin belongs to the penicillin group of beta-lactam antibiotics, it is able to penetrate Gram-positive and some Gram-negative bacteria. It acts as a competitive inhibitor of the enzyme transpeptidase, which is needed by bacteria to make their cell walls. Inhibition of cell wall synthesis ultimately leads to cell lysis. Gentamicin targets mainly Gram-negative bacterium and inhibits protein synthesis. Metronidazole is a nitroimidazole antibiotic used particularly for anaerobic bacteria (particularly from the genus clostridium and bacteroides) and protozoa. Once metronidazole is taken up by the microorganisms, it is non-enzymatically reduced by reacting with reduced ferredoxin, which is generated by pyruvate oxidoreductase. Many of the reduced nitroso intermediates will form sulfinamides and thioether linkages with cysteine-bearing enzymes, thereby deactivating these critical enzymes. Paromomycin sulfate is a protein synthesis inhibitor and binds directly to the 16S rRNA. It is a broad spectrum antibiotic which is soluble in water.

Rates of application of ampicillin, gentamicin and metronidazole were measured twice daily at 10.00 and 16.00 for 6 days until the end of the experiment. Paromomycin sulfate was applied only twice on the first day owing to the cost of this antibiotic.

Sterile surgical gloves were worn at all times to avoid contamination. Samples were taken either when the corals had less than half their remaining tissue on the nubbins or at the end of the experiment. Samples were placed in Falcon tubes underwater and sealed. The water was then replaced with 100% ethanol and stored at −20°C until extraction.

### (a) PCR and denaturing gradient gel electrophoresis

Extraction, PCR and denaturing gradient gel electrophoresis were undertaken as described in [16]. DNA was extracted from all samples using QIAGEN DNeasy blood and tissue kits, and bacterial 16S rRNA gene diversity was amplified using primers 357F and 518R. PCR protocol was as in [17]. Ciliate 18S rRNA gene diversity was amplified using primers CilF and CilDGGE-r. PCR protocol was as in [17]. For each of the above primer pairs, 30 µl PCR mixtures containing 1.5 mM MgCl_2_, 0.2 mM dNTP (Promega), 0.5 mM of each primer, 2.5 Ul of Taq DNA polymerase (QBiogene), incubation buffer and 20 ng of template DNA [17]. DGGE was performed as in [17] using the D-Code universal mutation detection system (Bio-Rad). PCR products were resolved on 10% (w/v) polyacrylamide gels for bacterial 16S rRNA gene diversity and 8% (w/v) for ciliate diversity. Bands of interest (those which explained the greatest differences/similarities between samples) were excised from DGGE gels, re-amplified with the same original primers, labelled using a big dye (Applied Biosystems) transformation sequence kit and sent to Genevision (Newcastle University, UK) for sequencing.

### (b) Clone libraries and amplified ribosomal DNA restriction analysis screening

As all replicates within samples showed no significant differences in relation to their DGGE profiles, a random subset of only *n* = 3 replicates per treatment were further analysed using Clone Libraries. Almost complete 16S rRNA gene fragments were amplified from the DNA extracted using the ‘universal’ eubacterial 16S rRNA gene primers 27F and 1542R. PCR protocols were as in [27,36]. Amplified products, purified using the Qiagen PCR purification kit, were inserted into the pGEM-T vector system (Promega) and transformed into *Escherichia coli* JM109 cells. A total of 392 clones containing the 16S rRNA gene inserts were randomly selected from each sample/replicate, and boiled lysates were prepared as in [17]. PCR protocols were as in [17]. The products were then digested with the restriction enzymes *Hae*III and *Rsa*I (Promega; 4 µg of PCR product, 2 µl of restriction buffer, 0.2 µl of bovine serum albumin, 0.07 µl of *Hae*III, 0.1 µl of *Rsa*I and made up to 20 µl with sigma water for 2 h at 37°C then 10 min at 67°C). Restriction fragments were resolved by 3% agarose gel electrophoresis, visualized using a ultraviolet transilluminator and grouped based on restriction patterns. *n* = 10 representatives from each group were sequenced. Closest match of retrieved sequences was determined by RDP II similarity matching [37]. All sequences in this study have been deposited in GenBank and their unique accession numbers reported in the text.

### (c) Total bacterial abundance

To estimate bacterial abundance, 1000 µl of tissue slurry was filtered through a 0.22 µm black polycarbonate filter and fixed with 100 µl of paraformaldehyde until analysis. These filters were stained with 100 μl DAPI solution (final concentration 5 μg ml^−1^) for 10 min, rinsed with phosphate-buffered solution, and viewed under epifluorescence microscopy (Nikon UK Ltd, Surrey, UK) at 1000× magnification using a DAPI-specific filter set. Counts on 50 fields of view were taken using an automatic cell counter (Cell C). The parameters were set to exclude any objects smaller than 0.03 μm^2^ and anything larger than 0.7 μm^2^. Counts were scaled up to the total area of the filter and calculated to give total bacterial abundance per volume of tissue on the diseased corals (cells cm^3^). Total amount of diseased tissue rather than complete coral nubbin surface area was used to account for the varying amount of tissue on the diseased samples as this could not be standardized at time of collection. All six nubbins were sampled for bacterial abundance with *n* = 3 subsamples taken from each coral and averaged to provide a cell density per sample.

### (d) Histology

Samples were collected as for microbial analysis; however, tissue samples were preserved with 5% paraformaldehyde for 24 h then stored in 100% EtOH until resin embedding in LR white (*r*). Survey sections of each tissue type were stained with the general DNA stain toluidine blue. The location of bacteria was recorded using fluorescent *in situ* hybridization (FISH). For FISH, samples were stained and sectioned following the protocols in [38]. Oligonucleotide probes were purchased from Interactiva (http://www.interactiva.de) with an aminolink C6/MMT at the 5′ end. Four probes were used: the ‘universal’ eubacterial probes EUB338, EUB338-II, EUB338-III and the ‘non-sense probe’ NONEUB. The three eubacterial probes were used in an equimolar mix (EUBMIX), and the NONEUB probe was used singly. Samples of pure cultured *E. coli* were run alongside each section and for each staining protocol as a positive stain. Sections were viewed under epiflourescence microscopy with an FITC-specific filter block (Nikon UK Ltd, Surrey, UK) and images recorded using an integrating camera (model JVC KY-SSSB: Foster Findlay and Associates, Newcastle upon Tyne, UK). Further histological samples were analysed for signs of apoptosis using ISEL of fragmented DNA (*in situ* apoptosis detection kit S7101 Chemicon International, Inc. USA) as per [33], whereby apoptotic cells are brown. The stain nigrosin [39] was used for evaluating the extent of mass tissue necrosis, necrotic cells appear black/brown in coloration.

### (e) Statistical analysis

Because the same experimental subjects (coral nubbins) were followed throughout the experiment, a two-way repeated-measure ANOVA was conducted to test the effect of each drug on WBD progression rates over time. The analysis was conducted with Statistica for Windows v. 6. An ANOSIM was conducted to test differences in 16S rRNA gene bacterial assemblage and 18S rRNA gene ciliate assemblage. Percentage similarity (SIMPER) was performed to determine which ribotypes better explained differences and/or similarities between sample types. Patterns of the 16S rRNA gene bacterial assemblages were represented on a multidimensional scaling plot.

## Supplementary Material

Timelapse video showing a diverse community of ciliates eating apparently healthy coral tissue

## Supplementary Material

Table S1
