# Supplementary figures and images for: Experimental antibiotic treatment identifies potential pathogens of white band disease in the endangered Caribbean coral Acropora cervicornis
Source: Proc Biol Sci. 2014 Aug 7;281(1788):20140094. doi: 10.1098/rspb.2014.0094 (PMC4083779; doi:10.1098/rspb.2014.0094)

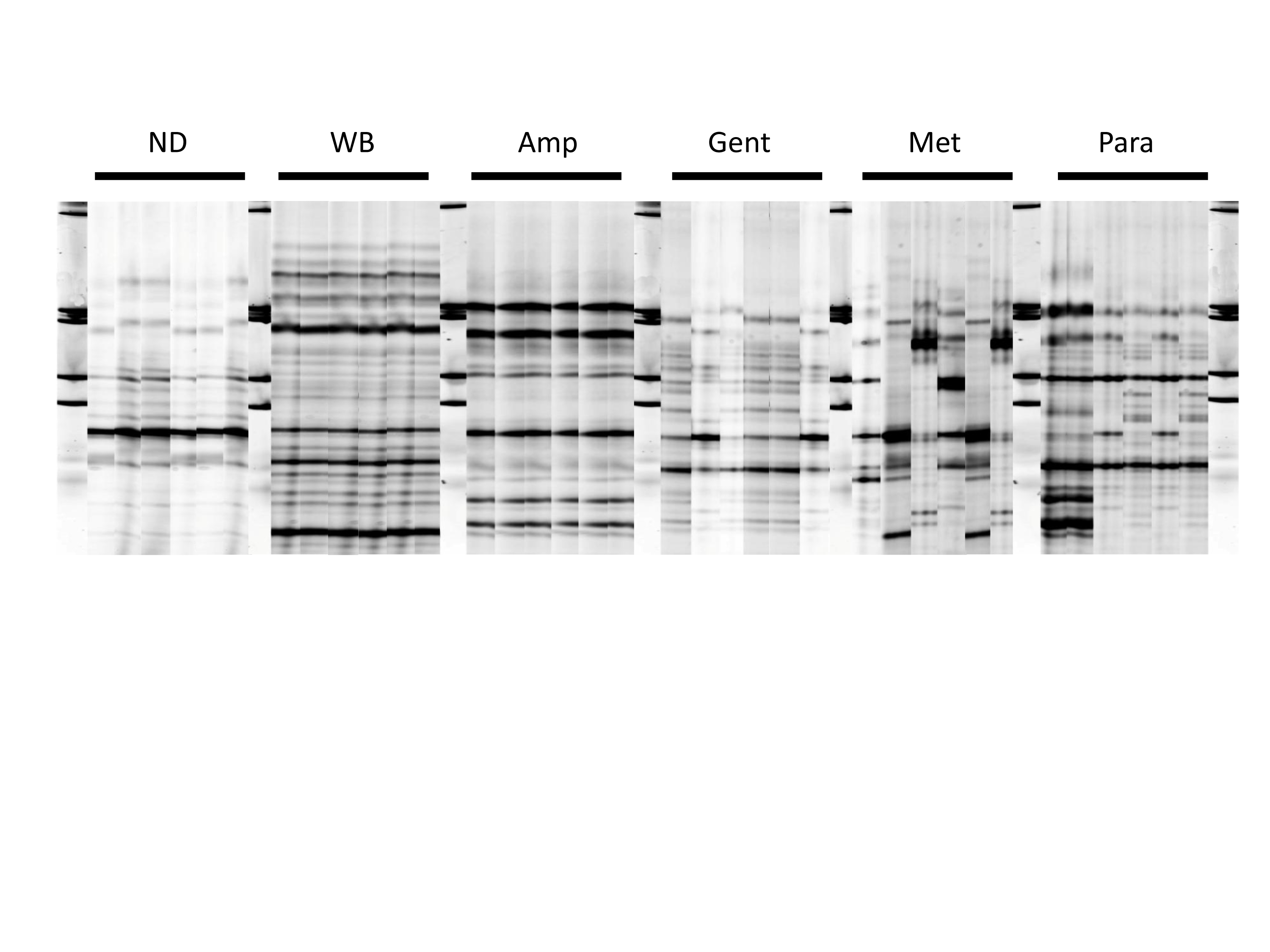

Supplement: Timelapse video showing a diverse community of ciliates eating apparently healthy coral tissue [file rspb20140094supp1.tif]
