# Supplementary material for: Experimental antibiotic treatment identifies potential pathogens of white band disease in the endangered Caribbean coral Acropora cervicornis
Source: Proc Biol Sci. 2014 Aug 7;281(1788):20140094. doi: 10.1098/rspb.2014.0094 (PMC4083779; doi:10.1098/rspb.2014.0094)
Supplement: Table S1 [file rspb20140094supp2.docx]

**Table S1.** Heatmap table summarizing the relative abundance (%) of dominant bacterial sequence affiliations of 16S rRNA gene clone libraries. Potential pathogens identified as those present consistently in all samples of diseased tissues (progressing lesions; WB, Gent and Met) but absent from non-progressive lesions (ND, Amp and Para). ID = identification of bacteria; for 100% sequence matches the bacteria were identified to species level, for greater than or equal to 98.5% similarity bacteria were identified to genus and for less than 98.5% similarity bacteria were identified to class.

| **Unique Accession No.** | **ID** | **Type** | **Closest match** | **ND** | **ND** | **ND** | **WB** | **WB** | **WB** | **Amp** | **Amp** | **Amp** | **Gent** | **Gent** | **Gent** | **Met** | **Met** | **Met** | **Para** | **Para** | **Para** |
| --- | --- | --- | --- | --- | --- | --- | --- | --- | --- | --- | --- | --- | --- | --- | --- | --- | --- | --- | --- | --- | --- |
| KC736995 | *Oceanicola* | [Gram-negative](http://en.wikipedia.org/wiki/Gram-negative) | NR_043915 | 0 | 0 | 0 | 2 | 2 | 3 | 2 | 2 | 3 | 0 | 0 | 0 | 1 | 0 | 1 | 0 | 0 | 0 |
| KC736996 | *Sandarakinotalea* | [Gram-negative](http://en.wikipedia.org/wiki/Gram-negative) | NR_041300 | 0 | 0 | 0 | 3 | 3 | 4 | 5 | 3 | 3 | 0 | 0 | 0 | 1 | 1 | 1 | 4 | 3 | 4 |
| KC736997 | *Sphingopyxis litoris* | [Gram-negative](http://en.wikipedia.org/wiki/Gram-negative) | NR_043955 | 2 | 2 | 3 | 2 | 2 | 2 | 4 | 2 | 2 | 0 | 0 | 0 | 2 | 2 | 1 | 0 | 0 | 0 |
| KC736998 | *Anaeroplasma bactoclasticum* | [Mollicutes](http://en.wikipedia.org/wiki/Mollicutes) | NR_044675 | 0 | 0 | 0 | 3 | 3 | 4 | 2 | 3 | 3 | 5 | 4 | 5 | 1 | 2 | 2 | 5 | 4 | 4 |
| KC736999 | *Cyanobacteria* | Cyanobacteria | AF544885 | 4 | 4 | 5 | 0 | 0 | 0 | 0 | 0 | 0 | 3 | 4 | 4 | 0 | 2 | 2 | 3 | 3 | 4 |
| KC737000 | *Pseudoalteromonas* | [Gram-negative](http://en.wikipedia.org/wiki/Gram-negative) | GQ406782 | 2 | 3 | 4 | 4 | 0 | 0 | 2 | 0 | 0 | 0 | 0 | 0 | 4 | 3 | 2 | 0 | 0 | 0 |
| KC737001 | *Kocuria* | Gram-positive | GQ391989 | 4 | 3 | 3 | 0 | 1 | 1 | 0 | 0 | 0 | 3 | 3 | 3 | 2 | 2 | 2 | 3 | 4 | 3 |
| KC737002 | [Thermoprotei](http://en.wikipedia.org/wiki/Thermoprotei) | [Archaea](http://en.wikipedia.org/wiki/Archaea) | NR_029316 | 2 | 3 | 2 | 2 | 3 | 2 | 4 | 3 | 3 | 4 | 4 | 5 | 4 | 2 | 2 | 5 | 3 | 3 |
| KC737003 | *Nautella italica* | [Gram-negative](http://en.wikipedia.org/wiki/Gram-negative) | NR_042673 | 0 | 3 | 0 | 3 | 3 | 2 | 2 | 4 | 4 | 0 | 0 | 0 | 1 | 2 | 2 | 4 | 4 | 4 |
| KC737004 | *Bacteroides coprophilus* | [Gram-negative](http://en.wikipedia.org/wiki/Gram-negative) | NR_041461 | 5 | 6 | 5 | 2 | 2 | 3 | 4 | 5 | 4 | 0 | 0 | 0 | 0 | 0 | 0 | 0 | 0 | 0 |
| KC737005 | *Rubrimonas cliftonensis* | [Gram-negative](http://en.wikipedia.org/wiki/Gram-negative) | NR_037114 | 3 | 5 | 3 | 4 | 3 | 4 | 5 | 6 | 5 | 0 | 0 | 0 | 3 | 3 | 3 | 0 | 0 | 0 |
| KC737006 | *Treponema lecithinolyticum* | [Gram-negative](http://en.wikipedia.org/wiki/Gram-negative) | NR_026247 | 4 | 4 | 3 | 3 | 3 | 3 | 4 | 4 | 5 | 0 | 0 | 0 | 4 | 2 | 2 | 0 | 0 | 0 |
| KC737007 | *Clostridium ramosum* | Gram-positive | NR_029247 | 3 | 4 | 4 | 3 | 3 | 4 | 0 | 0 | 0 | 2 | 4 | 4 | 0 | 0 | 0 | 2 | 3 | 2 |
| KC737008 | *Chelonobacter* | [Gram-negative](http://en.wikipedia.org/wiki/Gram-negative) | NR_044466 | 0 | 0 | 1 | 2 | 1 | 2 | 2 | 3 | 2 | 0 | 0 | 0 | 1 | 0 | 0 | 0 | 0 | 0 |
| KC737009 | *Geobacillus* | Gram-positive | NR_028789 | 1 | 0 | 1 | 1 | 0 | 0 | 0 | 0 | 0 | 1 | 3 | 3 | 0 | 0 | 0 | 3 | 4 | 3 |
| KC737010 | *Parachlamydia acanthamoebae* | [Gram-negative](http://en.wikipedia.org/wiki/Gram-negative) | NR_026357 | 0 | 0 | 0 | 1 | 2 | 2 | 0 | 0 | 0 | 0 | 1 | 0 | 3 | 3 | 4 | 0 | 0 | 0 |
| KC737011 | *Spiroplasma* | [Mollicutes](http://en.wikipedia.org/wiki/Mollicutes) | NR_036849 | 5 | 4 | 4 | 2 | 3 | 4 | 2 | 3 | 3 | 5 | 4 | 5 | 1 | 2 | 3 | 5 | 4 | 4 |
| KC737012 | *Mycoplasma* | [Mollicutes](http://en.wikipedia.org/wiki/Mollicutes) | NR_041845 | 2 | 2 | 3 | 2 | 2 | 3 | 2 | 2 | 3 | 5 | 6 | 6 | 2 | 3 | 4 | 4 | 4 | 4 |
| KC737013 | *Lactobacillus reuteri* | Gram-positive | NR_025911 | 1 | 3 | 3 | 1 | 1 | 2 | 0 | 0 | 0 | 0 | 0 | 0 | 1 | 2 | 1 | 1 | 0 | 0 |
| KC737014 | [Mollicutes](http://en.wikipedia.org/wiki/Mollicutes) | [Mollicutes](http://en.wikipedia.org/wiki/Mollicutes) | NR_041845 | 3 | 2 | 2 | 1 | 2 | 2 | 2 | 3 | 3 | 6 | 5 | 5 | 2 | 3 | 3 | 0 | 3 | 3 |
| KC737015 | *Photobacterium aplysiae* | [Gram-negative](http://en.wikipedia.org/wiki/Gram-negative) | NR_043188 | 0 | 0 | 0 | 4 | 4 | 4 | 2 | 3 | 4 | 0 | 0 | 0 | 1 | 1 | 1 | 0 | 0 | 0 |
| KC737016 | *Geothrix* | Unknown | NR_036779 | 4 | 3 | 3 | 0 | 0 | 1 | 0 | 1 | 1 | 4 | 4 | 5 | 4 | 3 | 4 | 3 | 3 | 3 |
| KC737017 | *Comamonas* | [Gram-negative](http://en.wikipedia.org/wiki/Gram-negative) | NR_044039 | 0 | 0 | 0 | 3 | 4 | 4 | 2 | 3 | 3 | 0 | 0 | 0 | 1 | 2 | 0 | 0 | 0 | 0 |
| KC737018 | *Leptobacterium flavescens* | [Gram-negative](http://en.wikipedia.org/wiki/Gram-negative) | NR_041638 | 0 | 1 | 0 | 0 | 1 | 0 | 5 | 3 | 4 | 0 | 0 | 0 | 2 | 1 | 1 | 0 | 0 | 0 |
| KC737019 | *Pseudoalteromonas* | [Gram-negative](http://en.wikipedia.org/wiki/Gram-negative) | GQ391963 | 4 | 2 | 3 | 1 | 2 | 1 | 3 | 2 | 2 | 0 | 0 | 0 | 3 | 3 | 1 | 4 | 4 | 3 |
| KC737020 | [Halobacteria](http://en.wikipedia.org/wiki/Halobacteria) | [Archaea](http://en.wikipedia.org/wiki/Archaea) | NR_028199 | 0 | 0 | 0 | 3 | 4 | 3 | 2 | 3 | 2 | 5 | 5 | 6 | 3 | 3 | 3 | 2 | 3 | 3 |
| KC737021 | *Treponema* | [Gram-negative](http://en.wikipedia.org/wiki/Gram-negative) | NR_029296 | 5 | 2 | 2 | 0 | 0 | 0 | 0 | 0 | 0 | 0 | 0 | 0 | 3 | 3 | 2 | 0 | 0 | 0 |
| KC737022 | *Asteroleplasma* | [Mollicutes](http://en.wikipedia.org/wiki/Mollicutes) | NR_044657 | 0 | 0 | 0 | 3 | 3 | 4 | 5 | 4 | 4 | 6 | 5 | 5 | 1 | 2 | 2 | 5 | 5 | 4 |
| KC737023 | *Roseofilum reptotaenium* | Cyanobacteria | HM048872 | 4 | 2 | 3 | 0 | 0 | 0 | 0 | 0 | 0 | 3 | 5 | 4 | 2 | 3 | 3 | 1 | 2 | 2 |
| KC737024 | *Vibrio carchariae* | [Gram-negative](http://en.wikipedia.org/wiki/Gram-negative) | JF792090 | 0 | 0 | 0 | 2 | 2 | 3 | 0 | 0 | 0 | 1 | 2 | 1 | 1 | 2 | 2 | 0 | 0 | 0 |
| KC737025 | [Methanomicrobia](http://en.wikipedia.org/wiki/Methanomicrobia) | [Archaea](http://en.wikipedia.org/wiki/Archaea) | NR_028238 | 2 | 1 | 2 | 1 | 2 | 1 | 3 | 3 | 2 | 5 | 5 | 4 | 3 | 3 | 4 | 2 | 3 | 3 |
| KC737026 | *Lactobacillus suebicus* | Gram-positive | NR_042190 | 0 | 0 | 0 | 3 | 3 | 2 | 0 | 0 | 0 | 2 | 4 | 3 | 1 | 2 | 2 | 0 | 0 | 0 |
| KC737027 | [Alphaproteobacteria](http://en.wikipedia.org/wiki/Alphaproteobacteria) | [Gram-negative](http://en.wikipedia.org/wiki/Gram-negative) | AF544964 | 3 | 2 | 3 | 4 | 3 | 3 | 4 | 4 | 3 | 4 | 4 | 3 | 2 | 3 | 3 | 6 | 4 | 4 |
| KC737028 | *Nocardiopsis alba* | Gram-positive | JF792076 | 3 | 2 | 2 | 3 | 2 | 2 | 0 | 0 | 0 | 5 | 4 | 4 | 2 | 2 | 2 | 5 | 3 | 3 |
| KC737029 | *Tenacibaculum* | [Gram-negative](http://en.wikipedia.org/wiki/Gram-negative) | NR_044498 | 2 | 3 | 4 | 3 | 5 | 2 | 4 | 4 | 3 | 0 | 0 | 0 | 3 | 3 | 2 | 0 | 0 | 0 |
| KC737030 | *Nitrobacter* | [Gram-negative](http://en.wikipedia.org/wiki/Gram-negative) | NR_042449 | 5 | 2 | 3 | 0 | 0 | 0 | 0 | 0 | 0 | 0 | 0 | 0 | 0 | 0 | 0 | 0 | 0 | 0 |
| KC737031 | *Roseovarius crassostreae* | [Gram-negative](http://en.wikipedia.org/wiki/Gram-negative) | NR_041731 | 0 | 0 | 0 | 3 | 4 | 2 | 4 | 3 | 2 | 0 | 1 | 2 | 1 | 2 | 1 | 0 | 0 | 0 |
| KC737032 | *Bacillus* | Gram-positive | NR_028674 | 0 | 0 | 0 | 3 | 2 | 3 | 0 | 0 | 0 | 4 | 1 | 0 | 3 | 2 | 2 | 0 | 0 | 0 |
| KC737033 | *Acholeplasma* | [Mollicutes](http://en.wikipedia.org/wiki/Mollicutes) | NR_042958 | 4 | 3 | 2 | 0 | 0 | 0 | 0 | 0 | 0 | 3 | 2 | 2 | 1 | 2 | 2 | 5 | 4 | 5 |
| KC737034 | *Thalassobacter* | [Gram-negative](http://en.wikipedia.org/wiki/Gram-negative) | NR_044471 | 1 | 1 | 1 | 1 | 0 | 0 | 0 | 0 | 0 | 0 | 0 | 0 | 2 | 0 | 1 | 0 | 0 | 0 |
| KC737035 | Cyclobacterium | [Gram-negative](http://en.wikipedia.org/wiki/Gram-negative) | NR_043903 | 0 | 0 | 0 | 3 | 2 | 2 | 4 | 4 | 3 | 0 | 0 | 0 | 2 | 1 | 2 | 0 | 0 | 0 |
| KC737036 | Betaproteobacteria | [Gram-negative](http://en.wikipedia.org/wiki/Gram-negative) | AY323190 | 0 | 0 | 0 | 2 | 2 | 2 | 4 | 3 | 2 | 0 | 0 | 0 | 5 | 3 | 4 | 4 | 3 | 3 |
| KC737037 | *Legionella* | [Gram-negative](http://en.wikipedia.org/wiki/Gram-negative) | NR_044963 | 2 | 2 | 4 | 2 | 2 | 2 | 3 | 4 | 3 | 0 | 0 | 0 | 2 | 3 | 2 | 0 | 0 | 0 |
| KC737038 | [Alphaproteobacteria](http://en.wikipedia.org/wiki/Alphaproteobacteria) | [Gram-negative](http://en.wikipedia.org/wiki/Gram-negative) | AY323140 | 3 | 3 | 2 | 3 | 2 | 2 | 3 | 3 | 2 | 5 | 3 | 3 | 5 | 3 | 3 | 5 | 5 | 4 |
| KC737039 | Actinobacteria | Gram-positive | JX549405 | 0 | 2 | 1 | 3 | 1 | 2 | 0 | 1 | 1 | 4 | 3 | 3 | 1 | 2 | 2 | 3 | 4 | 4 |
| KC737040 | *Vibrio rotiferianus* | [Gram-negative](http://en.wikipedia.org/wiki/Gram-negative) | JF792073 | 4 | 3 | 2 | 0 | 0 | 0 | 2 | 0 | 0 | 0 | 0 | 0 | 0 | 1 | 2 | 4 | 3 | 4 |
| KC737041 | [Alphaproteobacteria](http://en.wikipedia.org/wiki/Alphaproteobacteria) | [Gram-negative](http://en.wikipedia.org/wiki/Gram-negative) | AY323132 | 0 | 2 | 2 | 3 | 3 | 2 | 4 | 3 | 4 | 5 | 4 | 4 | 1 | 2 | 0 | 3 | 3 | 2 |
| KC737042 | *Pseudoalteromonas* | [Gram-negative](http://en.wikipedia.org/wiki/Gram-negative) | AB675038 | 3 | 2 | 2 | 0 | 1 | 0 | 1 | 0 | 0 | 0 | 0 | 0 | 3 | 2 | 2 | 4 | 3 | 3 |
| KC737043 | *Streptococcus* | Gram-positive | NR_042777 | 2 | 3 | 3 | 2 | 1 | 2 | 0 | 1 | 3 | 5 | 4 | 4 | 3 | 2 | 3 | 0 | 3 | 3 |
| KC737044 | *Mycoplasma* | [Mollicutes](http://en.wikipedia.org/wiki/Mollicutes) | NR_041845 | 4 | 5 | 5 | 0 | 0 | 0 | 0 | 0 | 0 | 2 | 3 | 3 | 0 | 0 | 2 | 4 | 2 | 4 |
| KC737045 | *Pyrobaculum* | [Archaea](http://en.wikipedia.org/wiki/Archaea) | NR_044615 | 0 | 1 | 0 | 3 | 2 | 2 | 0 | 2 | 4 | 3 | 3 | 4 | 1 | 1 | 2 | 1 | 2 | 2 |
| KC737046 | *Sphingobium* | [Gram-negative](http://en.wikipedia.org/wiki/Gram-negative) | NR_042479 | 0 | 2 | 3 | 3 | 4 | 2 | 3 | 3 | 5 | 0 | 0 | 0 | 3 | 3 | 2 | 0 | 2 | 3 |
| KC737047 | *Roseobacter litoralis* | [Gram-negative](http://en.wikipedia.org/wiki/Gram-negative) | NR_027593 | 4 | 3 | 2 | 0 | 0 | 0 | 0 | 0 | 0 | 0 | 0 | 0 | 2 | 1 | 1 | 0 | 0 | 0 |
| Total Number of Individual Sequences Retrieved from Specific Samples | | | | 32 | 37 | 35 | 41 | 41 | 40 | 32 | 34 | 33 | 26 | 28 | 26 | 46 | 45 | 46 | 28 | 30 | 30 |
